# An Exploratory Study of the Association between Housing Price Trends and Antidepressant Use in Taiwan: A 10-Year Population-Based Study

**DOI:** 10.3390/ijerph18094839

**Published:** 2021-04-30

**Authors:** Chen-Yin Lee, Pao-Huan Chen, Yen-Kuang Lin

**Affiliations:** 1 Department of Applied Foreign Languages, Mingdao University, ChangHua 52345, Taiwan; Inin1210@gmail.com; 2 Department of Psychiatry, Taipei Medical University Hospital, Taipei 110, Taiwan; b8601115@tmu.edu.tw; 3 Department of Psychiatry, School of Medicine, College of Medicine, Taipei Medical University, Taipei 110, Taiwan; 4 Statistics Center, Taipei Medical University, Taipei 110, Taiwan

**Keywords:** housing prices, mental disorder, antidepressant, economic movement

## Abstract

This study examined the relationship between various economic indexes and incidences of antidepressant prescriptions during 2001–2011 using the National Health Insurance Research Database (NHIRD). As of 2007, approximately 98.4% of Taiwanese people were enrolled in the NHIRD. In total, 531,281 records identified as antidepressant prescriptions were collected. Furthermore, 2556 quarterly observations from the Taiwan Housing Index (THI) and Executive Yuan were retrieved. We examined the association between the housing index and antidepressant prescription incidence. During the 10-year follow-up period, a higher incidence of antidepressant prescriptions was associated with the local maximum housing index. The relative risk of being prescribed antidepressant increased by 13.3% (95% confidence interval (CI): 1.01~1.27) when the THI reached a peak. For the low-income subgroup, the relative risk of being prescribed antidepressants increased by 28% during the high season of the THI. We also stratified the study sample on the basis of their sex, age, and urbanization levels. Both sexes followed similar patterns. During 2001–2011, although rising economic indexes may have increased incomes and stimulated the housing market, the compromise of public mental health could be a cost people have to pay additional attention to.

## 1. Introduction

### 1.1. Mental Disorders and Socioeconomic Stress

Mental disorders are a general diagnostic category for the dysregulation of emotions, thought processes, and behaviors, as defined by the Diagnostic and Statistical Manual of Mental Disorders, Fourth Edition, of the American Psychiatric Association. Mental disorders are highly prevalent in most countries, and the disease can progress over a person’s lifetime [1,2]. In the United States, approximately 8.1% of Americans aged at least 20 years experienced depression in a given two-week period [3]. In 2013, mental disorders were the top ranked costliest conditions, at USD 201 billion [4]. According to the National Institute of Mental Health, in 2012, an estimated 43.7 million adults at least 18 years of age in the United States had a mental illness [5].

One of the earliest studies considering the lifetime prevalence rate of all psychiatric disorders in Taiwan was conducted in 1953, in which Lin reported that the prevalence rate of all psychiatric disorders was 0.94% on the basis of a census survey [6]. Subsequent research investigating psychiatric disorders revealed that the prevalence ranged from 0.9% to 24.2% [7,8]. More recently, according to a 20-year study in Taiwan, the prevalence of common mental disorders increased from 11.5% in 1990 to 23.8% in 2010 [9]. Fu et al. (2013) observed that the prevalence of common mental disorders fluctuated in parallel with unemployment, divorce, and suicide attempts.

A combination of biological, psychological, and environmental factors can explain the formation of mental disorders. In a systematic review of both cross-sectional and longitudinal studies, Goldman-Meller et al. (2010) observed that economic events could increase the risk of psychological disorders because of the causal relationships between financial strain and social and physical environment [10].

Despite some research indicating a negative relationship between aggregate economic measurements and the incidence of suicide attempts and violent behavior [11,12], the majority of studies have suggested that economic constraints affect social environments and therefore are linked to both treated and untreated disorders [13,14,15].

### 1.2. Housing Prices as a Proxy for Macroeconomics Conditions

Housing is the largest single form of wealth for homeowners. Regular housing price appreciations contribute to economic growth through increases in household purchases, which is consistent with the “wealth effect” concept. Mian, Sufi, and Trebbi (2015) used US household-level data to evaluate the impact of housing prices on economic activity and observed that the country’s economic output grew faster when its housing prices underwent sharp and deep corrections [16]. Housing price appreciations are positively correlated with economic growth because they directly and indirectly influence both the consumption and investment [17,18,19]. Aizenman et al. (2021) revealed that house price appreciations are positively associated with economic growth [17]. They further determined that if the housing market appreciated 5.4% per year for six years, and then depreciated sharply at 11% per three years in the five following years, the total contribution of a typical housing cycle to GDP growth would be approximately 3.8%. Zheng and Zhang (2012) revealed that there was a stable unidirectional Granger causality between housing investment and GDP in China. The direction of Granger causality extended from GDP to housing investment, and this causality remained at both national and regional levels, before and after the 1988 mass housing reform. By contrast, irregular housing price appreciations could be related to overinvestment and reduce economic output [19].

Catalano et al. (2011) suggested that economic environment indicators lead to an increased risk of mental disorders [20]. During a trajectory of positive economic growth, owning a house is considered not only a shelter for families but also capital for preserving wealth. The purpose of owning a house is twofold: daily living and capital preservation. When housing prices increases with a country’s GDP, investing in housing as a capital good is profitable and marketable. However, when a discrepancy exists between housing prices and affordability, problems related to housing that determine the different dimensions of social disadvantages such as deprivation may arise. In other words, the excessive expansion of the housing market might have a negative impact on wellbeing by preventing people from satisfying their basic needs and promoting positive social relationships through homeownership. This negative effect is markedly more detrimental to the poor and socially disadvantaged than to other social classes [21]. Social epidemiological studies have long considered housing to be a social determinant of mental health [22]. When the prospects of homeownership are hampered by financial constraints, the mental health of the prospective homeowners could be negatively affected [23]. The link between housing affordability and psychological conditions such as mental disorders is a topic of interest among researchers. However, research on effects of increasing housing prices on mental health is limited. Thus, we aimed to extend the literature by examining the correlation between economics and mental health through large-scale, evidence-based statistical analyses.

In a housing market analysis, macroeconomic factors were strongly correlated with housing price index [24]. Thus, we used housing price as a proxy for macroeconomic conditions. Compared with other major Asian markets, Taipei, the capital of Taiwan, has high housing prices [25], which have negatively affected housing affordability for Taipei residents. According to the latest Housing Demand Survey distributed by the Ministry of the Interior (2018), the ratio of housing prices to incomes was 14.44 for Taipei. Thus, the price shock generated in Taipei has produced a “ripple effect” [26] in other cities in Taiwan, namely Taichung and Kaohsiung.

### 1.3. Antidepressant Prescription as an Indicator of Mental Disorder

Notably, stress resulting from housing costs may result in not only severe mental health diagnoses but also minor mental health conditions, including sleeping problems, headaches, and chronic pain. Therefore, we considered antidepressant prescription to be an indicator of broad mental disorders. Furthermore, data on mental disorder severity were unavailable in the National Health Insurance Research Database (NHIRD). To further elucidate the severity of mental disorders and its correlation with time-dependent changes in housing prices, we used antidepressant prescription as a variable to develop a claim-based index [27]. More antidepressant prescriptions may indicate a higher prevalence of mental disorders. We hypothesized that sharp increases in housing prices were detrimental to mental health and that a drastic rise in housing prices would potentially be both a consequence and determinant of antidepressant prescriptions, which could be more effectively identified through quantifiability instead of fixed diagnoses.

### 1.4. Strength of Current Study

Housing market activities contribute to stable economic growth. However, housing prices continue to increase every year, putting pressure on populations that intend to purchase a house. The current study employed the longitudinal database of the universal, single-payer National Health Insurance (NHI) program in Taiwan. The longitudinal flow of antidepressant prescriptions was categorized by sex, income, and region, and their potential association with housing prices was investigated. The enormous number of prescriptions identified implies that the current study is highly representative of the study population.

## 2. Materials and Methods

### 2.1. Data Sources

Taiwan launched the NHI program in March 1995. NHI covers ambulatory and inpatient care, dental services, and prescription drugs. As of 2000, NHI covered for 21,400,826 residents, 96% of all Taiwan residents. In the interest of research development, the NHI continually tracks a cohort of randomly sampled participants, consisting of one million people, from among all insured residents. Thus, the current study used both ambulatory care data and inpatient expenditure data from the Longitudinal Health Insurance Database 2000 (LHID2000). The dataset includes the encrypted identification numbers of residents as well as data on sex, birth date, insurance amount, insured area, types and dates of healthcare services, and dates of inpatient admission and discharge.

### 2.2. Study Sample

#### 2.2.1. Therapeutic Indications for Antidepressant Use

To better identify antidepressant uses, we included both officially approved indications and off-label indications in clinical practice. Approved indications consisted of mood, anxiety, sleep, and adjustment disorders. Nonpsychiatric indications included chronic pain (headaches, back pain, neuropathic pain, and fibromyalgia), functional gastrointestinal disorders, dermatological disorders (urticaria, eczema, and pruritus), and urogenital dysfunction [28]. On the basis of a nationwide population-based study in Taiwan, the majority of indications for antidepressant prescriptions were mood disorders using selective serotonin reuptake inhibitors (SSRIs; 63.9%) and monoamine oxidase inhibitors (MAOIs; 56.2%), anxiety disorder treated with SSRIs (25.4%) and MAOIs (30%), and sleep disorders treated with trazodone (31.1%). Wu et al. found that the prevalence rates for off-label use of antidepressants prescribed in Taiwan for urogenital dysfunction, dermatological disorders, and functional gastrointestinal disorders were 5.6%, 8.7%, and 5.4% [28], respectively.

We identified participants who used antidepressants during the study period from 2001 to 2011. The prevalent antidepressant users were those who received at least one antidepressant prescription in a given year. We excluded people with an undetermined sex or age information from the NHI program. For analysis of antidepressant classes, if patients received more than one class of antidepressant within a given year, they were counted as one user of each class of antidepressant. Medications of interest were defined according to the Anatomical Therapeutic Chemical Classification System. These antidepressants and their ATC codes consisted of SSRIs: fluoxetine (N06AB03), sertraline (N06AB06), paroxetine (N06AB05), fluvoxamine (N06AB08), citalopram (N06AB04), and escitalopram (N06AB10); serotonin and norepinephrine reuptake inhibitor (SNRI): venlafaxine (N06AX16); tricyclic antidepressants (TCA): imipramine (N06AA02), amitriptyline (N06AA09), and doxepin (N06AA12); tetracyclic antidepressant (TeCA): nirtazapine (N06AX11); MAOI: moclobemide (N06AG02); and serotonin antagonist and reuptake inhibitor (SARI): trazodone (N06AX05). Notably, indications were not mutually exclusive, antidepressants could be prescribed for multiple indications, and thus they may appear multiple times in the database. Consequently, 531,281 prescriptions comprised the final sample. Furthermore, agomelatine, bupropion, reboxetine, and duloxetine are commonly prescribed in psychiatry currently. However, agomelatine has been available in Taiwan only since 2012. Therefore, the LHID for the study period 2000–2011 does not include information regarding agomelatine usage. While reboxetine was banned in 2006, bupropion and duloxetine were licensed within the study period but had relatively low prevalence, ranging from 0.2% to 0.7% [29,30]. Therefore, antidepressant prescriptions associated with agomelatine, bupropion, reboxetine, and duloxetine were not included.

#### 2.2.2. Proxy for Housing Price Trajectory

In the current study, we adopted the Sinyi house price index released by Sinyi Realty Company, one of the largest real estate brokerage firms in Taiwan. Based on the transaction database of existing houses, the Sinyi house price index was established for Taiwan as a whole and for four metropolises, including Taipei City, Taipei County, Taichung City, and Kaohsiung City [26,31]. To assess the additional effects of prolonged housing index fluctuations, some auxiliary variables were created. The changing effect was defined according to yearly changes in the average housing price index (yearly change). The peak effect was the effect of the local maximum of the housing index. To estimate the extreme effects of a housing index, an additional dummy variable “high season effect” was also created with a value of 1 for a housing index of >100, and 0 otherwise. In Taiwan, the median housing index during 2001–2006 was approximately 100. The covariates “peak effect” and “high season” were set to follow B-spine polynomial splines with five degrees of freedom and the “Taiwan Housing Index” (THI). The quarter effect was set as a dummy variable with the first quarter as the reference. Therefore, quarter estimates were made by comparing the target quarter with the first quarter. An indicator variable, SARS (severe acute respiratory syndrome), was also created and assigned a value of 1 if the counts were observed during the SARS outbreak period (26 April–5 July 2003) in Taiwan, and 0 otherwise. Finally, the 2008 financial crisis, and the opening price of the Taiwan Stock Market were included as covariates.

#### 2.2.3. Urbanization and Socioeconomic Status

In the current study, geographical factors such as urbanization were considered crucial factors affecting the impact of housing prices on mental health. The levels of urbanization are categorized into five strata based on population density, education level, age group, the proportion of agricultural workers, and the number of physicians [32]. To approximate participants’ socioeconomic status, insurance premium data were retrieved in addition to information on urbanization levels. Insurance premiums for individuals are calculated according to the monthly income reported to the NHI administration. Thus, we estimated income level based on insurance premiums and grouped into three categories (New Taiwan dollars (TWD) ≥ 21,000, 21,000–1007, and 1–1007). In the NHIRD, wage data are divided into 54 levels with 10 intervals. To avoid data manipulation, we used quantiles of the wage data of the total population to divide the study population into high-, middle- and low-income levels. High income was defined as a monthly wage of more than TWD 21,000 (80th percentile), middle income was between TWD 21,000 and TWD 1007 (80th to 30th percentile), and low income was lower than TWD 1007 (30th percentile). Because income distribution was positively skewed, participants were concentrated in the middle- to low-income area, resulting in more people below the high group than those in the high-income groups. Income was treated as socioeconomic status and measured once at baseline in 2000. Consequently, income was analyzed as a time-independent variable to reflect participants’ background information.

#### 2.2.4. Statistical Analysis

Statistical analyses were performed using SAS 9.4 (SAS Institute, Cary, NC, USA) and Statistical Environmental R 2.15. Variations in housing index timing were characterized by two chosen features: local maximum and global high season. The global high season consisted of points > 100, approximately the median of the housing index. The term “global” maximum is also known as the absolute maximum, the large overall value of the housing index over the entire study period. By contrast to the global maximum, the local maximum, which is defined as the “peak” in the current study, is a high value within a given interval. The distributed lag nonlinear model (DLNM) with a quasi-Poisson distribution was used to simultaneously estimate the nonlinear and delayed effects of the housing index on antidepressant prescriptions [33].

DLNMs employ a cross-basis function that describes a two-dimensional variation between the housing index and the number of antidepressant prescriptions along the dimensions of a housing index lag. Cross-basis functions are constructed by combining the basis functions for the two dimensions, which are produced by applying spline approximations. The choice of cross-basis functions for the housing index and number of antidepressant prescriptions are independent; therefore, the spline can be used for housing index fluctuations and the polynomial functions can be used for the lag. Polynomial-transformed DLNM models were used to analyze the nonlinear and delayed effects of housing prices. Relative risks (RRs) caused by housing prices were estimated using the cross-basis function in the DLNM models. Because the link between house prices and income has attracted considerable attention, we treated monthly income as a subgroup and conducted a subgroup analysis.

With these covariates, the model for expected antidepressant prescription count on time (t) is

(1) $$\mathrm{Log}\left(E_{y}\right)=\beta_{0}+\Sigma Polynomial\left(Peak,5;lag,5\right)+\Sigma Polynomial\left(Housing\;Index,,5;lag,5\right)+\Sigma Polynomial\left(High\;Season,5;lag,5\right)+\mathrm{Linear}\;\mathrm{Trend}+\mathrm{Quarter}+\mathrm{Yearly}\;\mathrm{Change}+\mathrm{SARS}+\mathrm{Stock}\;\mathrm{price}+\mathrm{Financial}\;\mathrm{Crisis}$$

where y is the observed antidepressant prescription at time t; *β*_0_ is the intercept; Polynomial (.) indicates a polynomial transformation; five degrees of freedom (df) for peak, and 5 df for lagged effect. The fluctuation effect was defined by the yearly change in the average housing price index (Yearly Change) whereas the linear trajectory of antidepressants was depicted with Linear Trend. The quarter effect was set as a dummy variable with the first quarter as the reference quarter. SARS was also generated and assigned a value of 1 if the antidepressant counts were observed during the SARS outbreak period (26 April~5 July 2003) and 0 otherwise. We also included dummy coding for the 2008 financial crisis, and the opening price of the Taiwan Stock Market in the distributed lag linear model. The hyperparameters for DLNM such as degrees of freedom, maximum lag day, and cross-basis type are flexible, and the choices for DLNM can be selected according to the best model fit [34]. Akaike’s information criterion for quasi-Poisson (Q-AIC) was used to determine the degree of freedom for the housing index and lag that was used to predicted number of prescriptions [35].

In the current study, each hypothesis was tested using two-sided alternatives with a significance level of 0.05. The standardized mean difference (SMD) was used to measure distances between groups. For continuous variables, the distance between two group means was calculated as follows:

$${\mathrm{SMD}}_{\mathrm{continuous}}=(\frac{{\bar{x}}_{group1}-{\bar{x}}_{group2}}{\sqrt{\frac{s_{group1}^{2}+s_{group2}^{2}}{2}}})$$

For categorical variables, the standardized difference was used to compare the difference in means between units of the pooled standard deviation (Austin, 2009). For a binary case, a proportion of 1 is the mean of that level after the variable is dummy coded. The variance is the proportion divided by 1 minus the proportion, or *p*/(1-*p*). Thus, the SMD is the difference in mean outcome between the group divided by the pooled standard deviation of the proportion, expressed as

$${\mathrm{SMD}}_{\mathrm{categorical}}=(\frac{{\hat{p}}_{group1}-{\hat{p}}_{group2}}{\sqrt{\frac{{\hat{p}}_{group1}\left(1-{\hat{p}}_{group1}\right)+{\hat{p}}_{group2}\left(1-{\hat{p}}_{group2}\right)}{2}}})$$

The concept can be generalized to multinomial variables with more than two levels [36]. This multinomial extension treats a single multinomial variable as multiple nonredundant dichotomous variables and use the Mahalanobis distance. The value of SMD can be understood as a Z-score of a standard normal distribution. Cohen (1988) suggested that effect size indices of 0.2, 0.5, and 0.8 can be used to represent small, medium, and large effect sizes, respectively [37]. If a standardized difference is 0.2, it indicates 15% nonoverlap in the two distributions, whereas a SMD of 0.5 indicates 33% nonoverlap [36]. The calculation of SMD in Table 1 was performed using the R package “tableone” version 0.12.0. The statistical test of the results is presented subsequently.

## 3. Results

In total, 531,281 antidepressant prescriptions were received from 2001 to 2011, approximately 4427 prescriptions per month during the study period. The average of Taiwanese housing prices (THI) throughout the study period was 122.32. The highest THI appeared in the second quarter of 2011 at a value of 170.13. Among all prescriptions, 329,752 were prescribed to females and 201,529 to males. Antidepressant were most commonly prescribed to patients aged 45–54 years (*n* = 118,522, 22.3%), those who lived in northern Taiwan (*n* = 248,098, 46.7%), those with an average monthly income of TWD 13,912 (standard deviation (SD) = 16,366) or USD 448 (SD = 527), and those with an average age of 52.86 (SD = 17.43) years. The annual change was 9.88 with an accumulation effect of 4.713 (SD = 10.79). Table 1 presents the descriptive statistics and the SMDs of all the variables.

**Figure 1:**
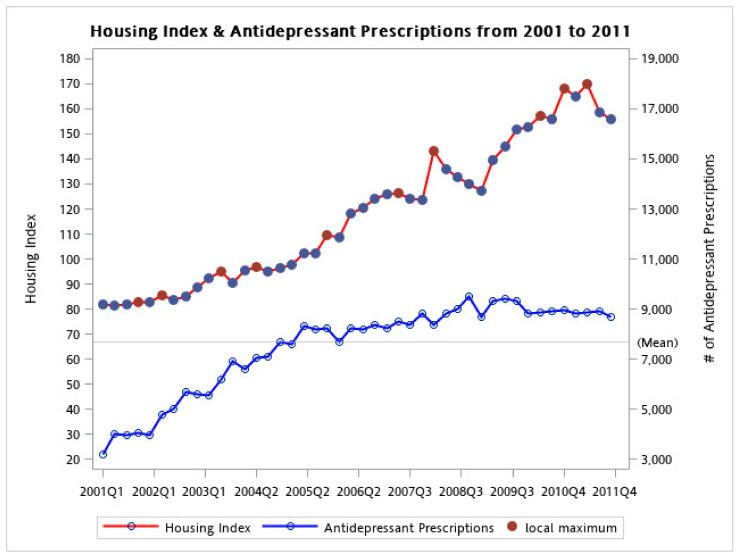
Growth trajectory of housing index and antidepressant prescriptions from 2001 to 2011. The figure shows the prevalence of antidepressant prescription along with the housing index, between the years 2001 and 2011. The housing indexes are presented with red dots while antidepressant prescriptions are marked in blue.

The THI gradually increased from 2001 to 2011 and appeared to plateau in 2011. The long-term trend for antidepressants followed a similar pattern to the THI, but appeared to plateau after 2008. Notably, when the THI reached a local maximum, the number of antidepressant prescriptions sharply increased followed by an increase in the THI (Figure 1). 

**Figure 2:**
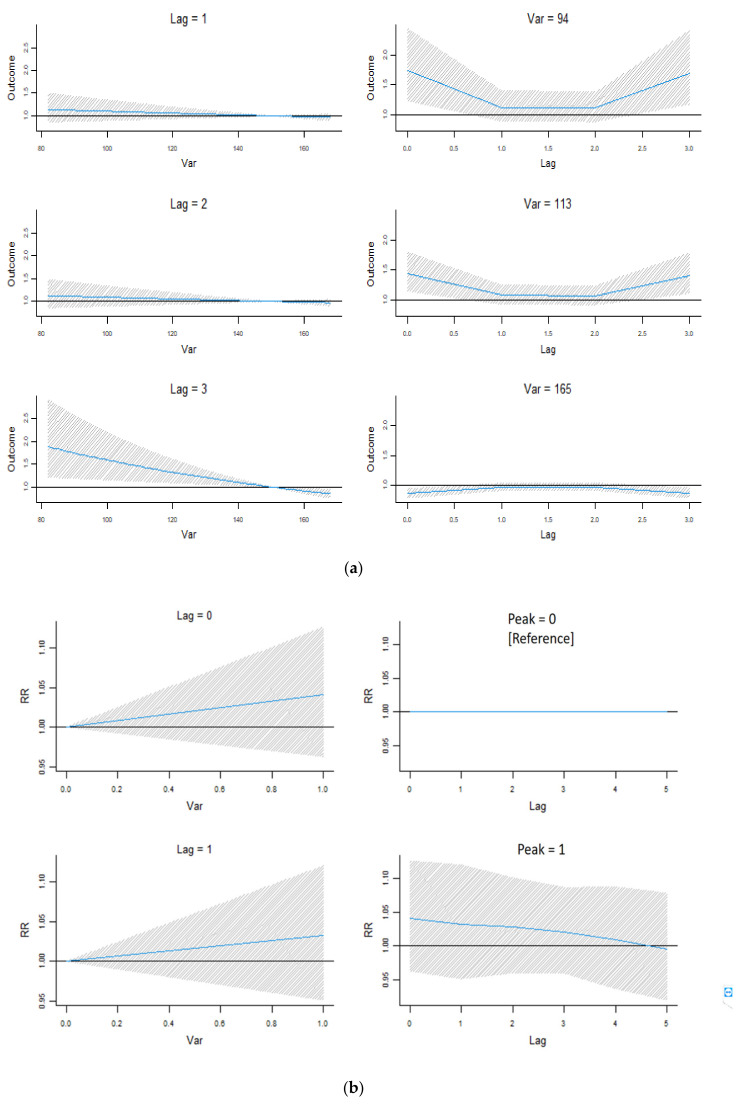
Coefficient plots of lag effects of housing prices: (**a**) interaction effects of the housing index and lag periods and (**b**) interaction effects of the peak in housing prices and lag periods.

The effects of the average housing index on antidepressant prescriptions were estimated using the DLNM after controlling for the quarter, SARS, financial crisis, stock, and long-term trends. Figure 2 presents the associations between the housing index and the relative risk (RR) of receiving a prescription. A higher probability of being prescribed an antidepressant was observed for housing indexes between 80 and 150 (Figure 2a in left) and at lag periods of 0, 1, and 3 (Figure 2a in right). The prolonged effects of a housing price peak were also observed. Figure 2b presents a visualization of the RRs with different lag periods at the peak in the housing market. 

Table 2 presents the results for the association between the housing index and antidepressant prescriptions. Among all recipients, a positive correlation was observed between the peak of housing prices and the number of prescriptions. The effect of the peak on the number of prescriptions was significant (*p* < 0.05).

**Figure 3:**
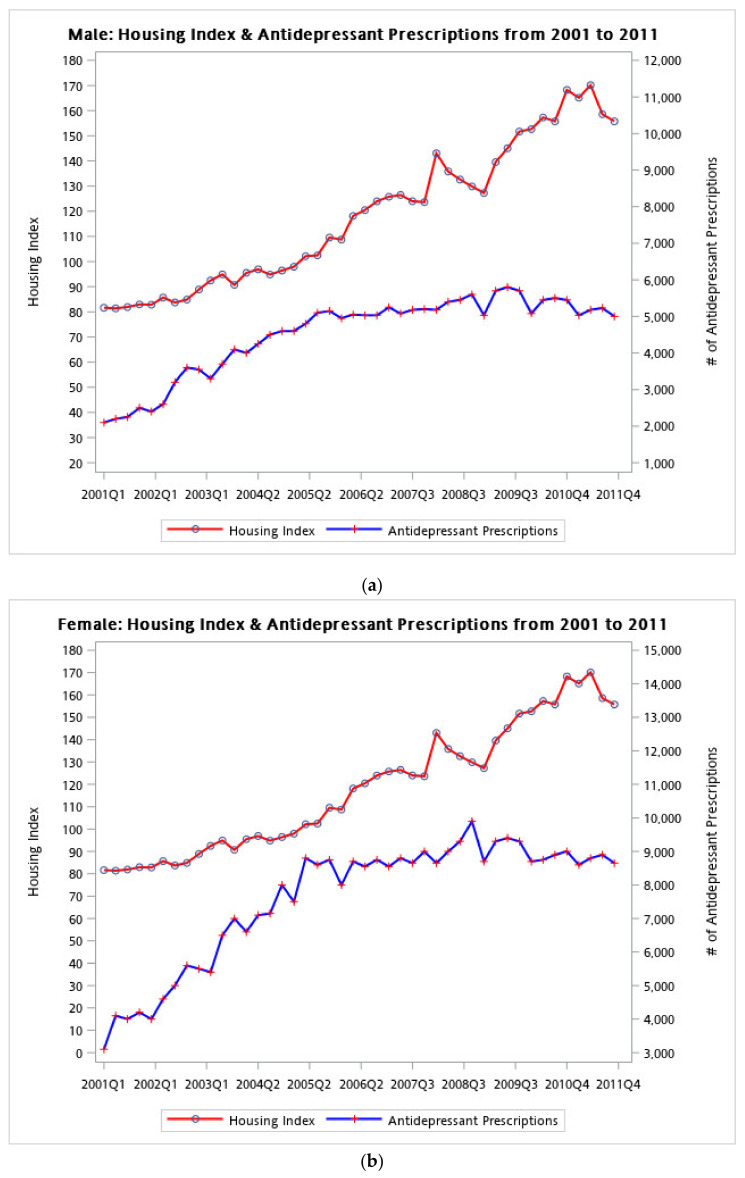
Line plot of housing indexes and antidepressant prescriptions stratified by sex. Housing indexes are presented as red dots, and antidepressant prescriptions are marked in blue. (**a**) Housing index and antidepressant prescriptions for male participants and (**b**) housing index and antidepressant prescriptions for female participants.

When the housing index was at its peak, antidepressant prescriptions had increased by 13.3% (RR = 1.13, 95% confidence interval (CI): 1.01–1.27). The RR of male participants being prescribed an antidepressant (RR = 1.082) was slightly higher than that of female participants (RR = 1.069). The lag effect of the peak was stronger than the peak’s current timing; that is, the lag 1 peak RR was 1.33 with a 95% CI of 1.02–1.74, implying that the risk of being prescribed an antidepressant could reach 74% or higher. After further stratification by sex, the linear effect appeared for female participants (RR: 1.10, 95% CI: 1.05–1.16) but not for male participants (Figure 3a,b).

When the housing market reached the high season, an increase in antidepressant prescriptions was observed for males and female participants (RR = 1.15, 1.38, respectively). In terms of the long-term linear trend in antidepressant prescriptions, a significant upward trend was observed for female participants. The association between the housing index and antidepressant prescriptions was further elucidated by stratifying antidepressant recipients into three income groups, namely, <TWD 1007, TWD 1007–TWD 21,000, and >TWD 21,000. The peak of the housing index had a positive effect on the low-income group (β = 0.171 *p* = 0.003), but not on the middle- or high-income group. When the housing market reached the high season, the low-income group exhibited a 24% increase in antidepressant prescriptions (*p* < 0.001) (Table 3).

**Figure 4:**
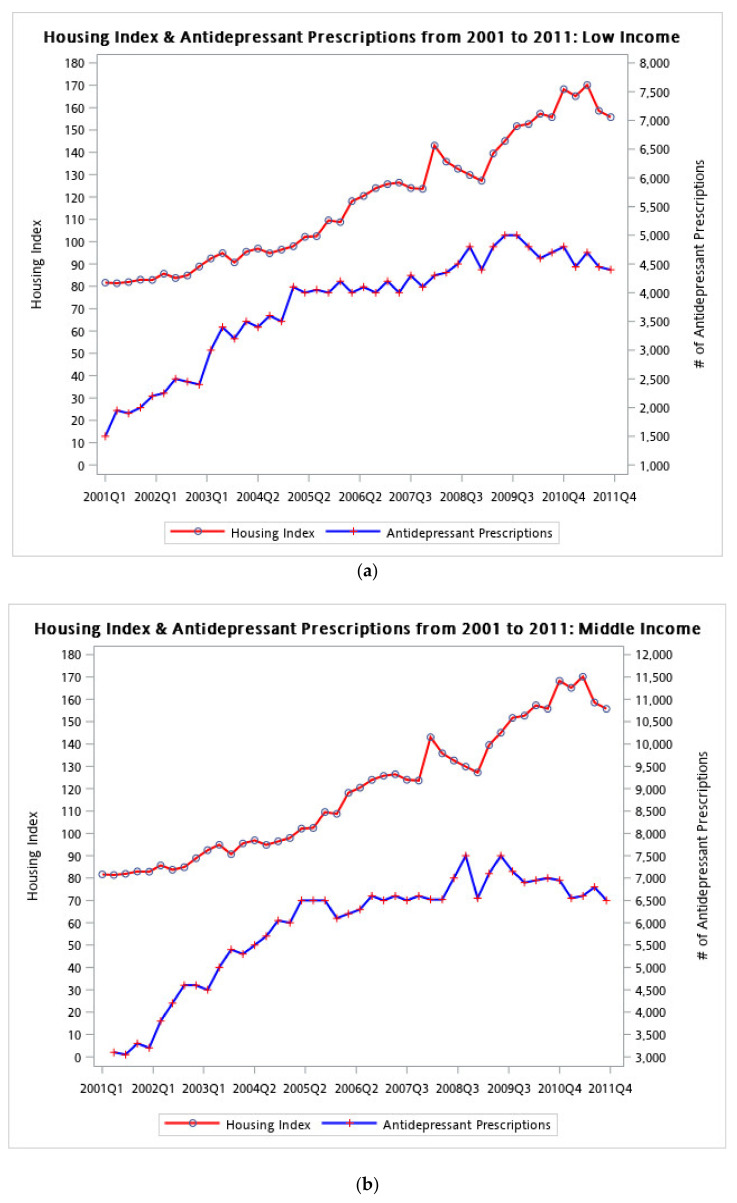

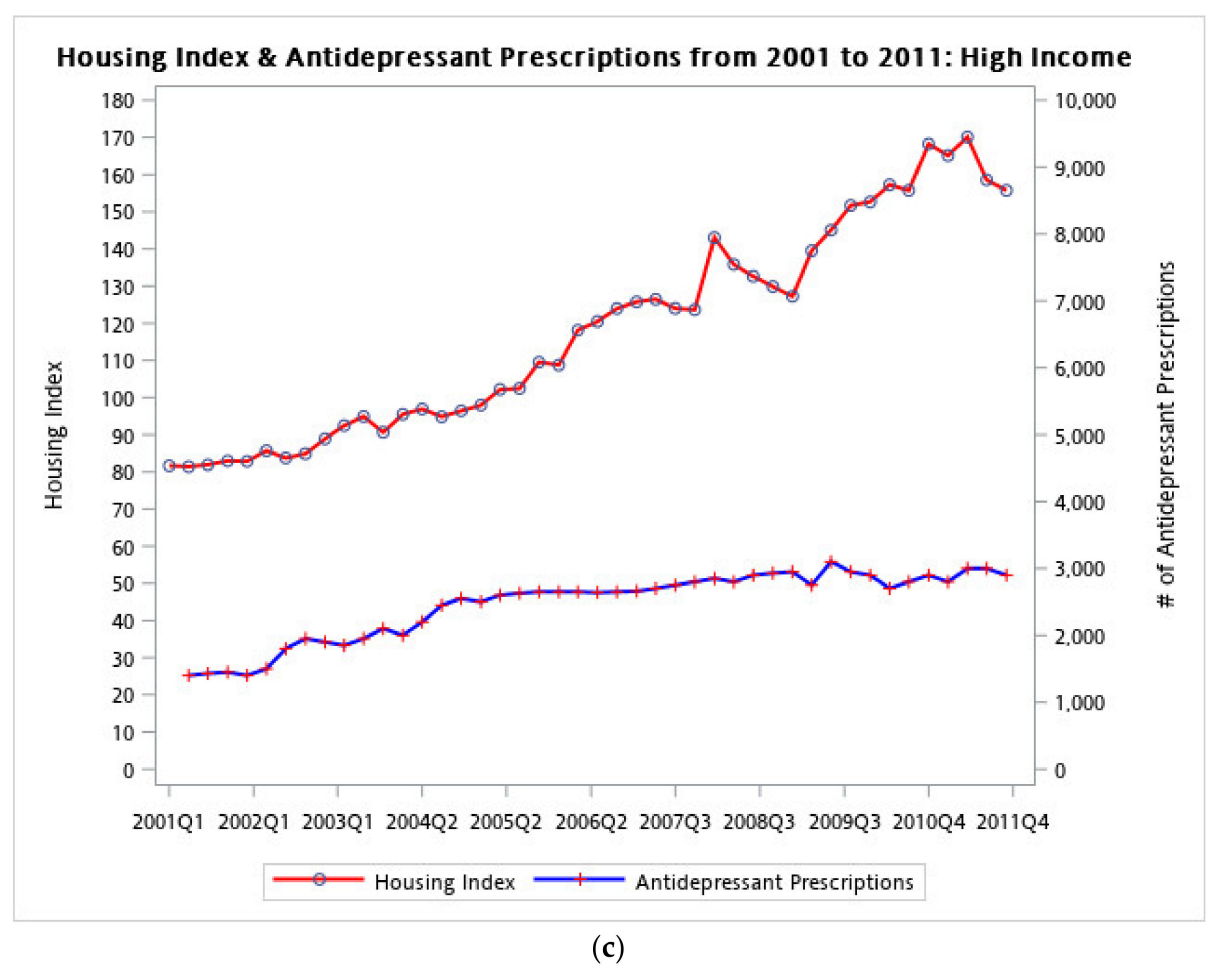
Line plot of housing indexes and antidepressant prescriptions stratified by income: (**a**) housing index and antidepressant prescriptions for low-income subgroup; (**b**) housing index and antidepressant prescriptions for middle-income subgroup; and (**c**) housing index and antidepressant prescriptions for the high-income subgroup.

The number of antidepressant prescriptions for the high-income group was less concordant with the housing index when compared to both the low- and middle-income groups. For the low- and middle-income group, the number of antidepressant prescription increased with the housing index from 2001 to 2008. By contrast, the number of antidepressant prescriptions for the high-income group fluctuated less with variations in the housing index (Figure 4a–c). We also conducted a subgroup analysis for different administrative districts and provided the results in a Supplementary Table S1. This result is consistent with those of the income subgroup examination because Kaohsiung has lower average incomes compared with Taipei and Taichung (Supplementary Table S1).

## 4. Discussion

This empirical study revealed that housing price variations had a strong unfavorable effect on antidepressant prescriptions. When the housing market was in the high season or at its peak, the probability of being prescribed an antidepressant was relatively high. The results showed that when the housing market reached a local maximum, the risk of being prescribed an antidepressant increased by 13.3%. The delayed peak effect resulted in a 33.3% increased probability being prescribed an antidepressant. When the housing market was in the high season, the number of prescribed antidepressants increased by 12.1%. Finally, a long-term increasing linear trend for antidepressant prescriptions was observed. We further explored these phenomena by stratifying prescription records into different income groups because stable income is a key factor determining housing affordability. The affordability aspect of housing problems has been thoroughly discussed in the literature. A reliable indicator of ability to pay is the housing expenditure-to-income ratio. In general, the ratio ranges from 20% to 50% [38,39,40,41]. In Taiwan, 30% of income spent on housing is the standard threshold for housing affordability, and households spending > 30% on housing are considered “housing cost burdened”. In Taiwan, the housing price-to-income ratio has rapidly increased to 17. That is, the ratio of the country’s GDP per capita to the cost of a typical upscale 100 m^2^ housing unit reached a record high of 17. The discrepancy between the growth in income and housing prices might gradually impose emotional and physical stress on potential buyers in Taiwan.

As the housing index increases, potential buyers may perceive the increase as a loss of wealth, possibly leading to stress and depression. However, those who already own a house may consider the increase to be an accumulation of wealth. This concept may explain the higher negative impact caused by housing index fluctuations on the low-income subgroup, but not on the higher income group [42,43]. Moreover, the peak of the housing price index was significant overall, and its high season effect was significant for both male and female participants. The high season effects were stronger for females than for male participants. One potential reason for the stronger effects on women is that women participate in the housing market more than males do.

Previous studies have demonstrated that people with relatively low socioeconomic status have a higher prevalence of mental disorders [44,45,46,47]. However, in our study, both the low and middle socioeconomic groups appeared to exhibit an increased prevalence of mental disorders over time. This paper can serve as a reference for policy makers responsible for housing investment decisions. The results herein suggest a positive relationship between irregular housing price fluctuation and mental health in the general population. The high cost of housing can be detrimental to low-income households compared with high-income households. Policy makers should acknowledge this possible negative impact and implement necessary interventions, including imposing property taxes to curb speculative demand and ease housing prices [48]. Nonrecourse mortgages should be limited because nonrecourse policies can cause housing prices to increase rapidly [49]. A nonrecourse mortgage gives borrowers limited liability with nonrecourse loans. Thus, borrowers may be more motivated to make speculative purchases, particularly during a market boom. Alternatively, in response to increasing concerns of housing affordability, several countries have accelerated their efforts of implementing small public housing programs that provide subsidized low-rent housing flats [21]. Taken together, our findings are consistent with literature, suggesting that governments prioritize a healthy housing market by ensuring a steady land supply and implementing demand-side intervention measures.

## 5. Conclusions

In this study, we observed that after a peak or high season in housing prices, an increase in antidepressant prescriptions occurred. Overall, low-income individuals and women had higher incidence rates of receiving antidepressant prescriptions. According to these findings, having a low income is a risk factor for being prescribed antidepressants regardless of sex. 

Finding affordable housing in Asian countries continues to draw attention and has always been challenging. Because our study indicates a potential link between housing prices and mental disorders, health policy makers should consider programs that minimize the social costs of increased depression or anxiety during times of socioeconomic crisis. For example, general psychoeducation to be provided for the population and general practitioners. In addition, in areas with high housing costs, additional mental health facilities can be established as a precaution.

During the study period, the psychiatric medical services were expanded through increases in the number of clinical facilities. For example, the number of psychiatric beds per 100,000 people increased from 22.5 to 29.9 from 2006 to 2010. The number of psychiatric outpatient departments increased from 274 to 348 [50]. A growing economy has various implications. For example, the positive impact of a growing economy may help governments reallocate resources to mental health services, which may alleviate disease burden. However, economic development may also lead to rising housing prices, which may negatively affect public mental health. Therefore, we extended the literature by examining the correlation between economics and mental health through evidence-based statistics.

Despite the strengths of our study, our findings must still be interpreted while considering several inherent limitations. First, antidepressant prescription records were based on claims data reported by both physicians and hospitals. However, antidepressants could also be obtained through pharmacies without filing insurance claims. Second, our study did not directly address whether the increase in housing prices could induce depression. Certain crucial socioeconomic and behavioral factors that affect mental health were unavailable in the dataset. Third, research has indicated an association between housing affordability and mental health in the United States [51]. We could have effectively explained and incorporated such an income housing cost discrepancy. However, because of the lack of data on the income–housing cost ratio at both regional and national levels, we could not evaluate housing affordability according to this ratio. 

In conclusion, the general observation that mental health is affected when housing prices increase much more quickly than wages do remains plausible. This research informs public health by identifying economic growth as a potential risk factor, particularly increases in housing price. The positive effect of economic growth is reduced in the disadvantaged population, for example, the lower-income group.

## Figures and Tables

**Table 1:** Descriptive statistics of antidepressant prescriptions (*n* = 531,281).

	All (*n* = 531,281)	Females (*n* = 329,752)	Males (*n* = 201,529)	SMD
*n*	531,281	329,752	201,529	
Age group, years (%)				0.146
<25	22,426 (4.2)	11,727 (3.6)	10,699 (5.3)	
25~34	67,397 (12.7)	37,902 (11.5)	29,495 (14.6)	
35~44	94,592 (17.8)	60,342 (18.3)	34,250 (17.0)	
45~54	118,522 (22.3)	75,229 (22.8)	43,293 (21.5)	
55~64	98,617 (18.6)	62,583 (19.0)	36,034 (17.9)	
65~74	59,046 (11.1)	39,191 (11.9)	19,855 (9.9)	
≥75	70,681 (13.3)	42,778 (13.0)	27,903 (13.8)	
Geography 2 (%)				0.111
Eastern	21,361 (4.0)	12,064 (3.7)	9297 (4.6)	
Central	82,986 (15.6)	52,176 (15.8)	30,810 (15.3)	
Northern	248,098 (46.7)	157,841 (47.9)	90,257 (44.8)	
Southern	171,838 (32.3)	104,582 (31.7)	67,256 (33.4)	
Monthly salary	13,912.34 ± 16,366.54	12,484.67 ± 14,411.60	16,272.26 ± 18,931.0	0.225
Administrative district (%)				0.094
Other	225,886 (42.5)	135,403 (41.1)	90,483 (44.9)	
Kaohsiung	71,580 (13.5)	43,478 (13.2)	28,102 (13.9)	
Taichung City	53,280 (10.0)	34,275 (10.4)	19,005 (9.4)	
Taipei City	107,691 (20.3)	69,327 (21.0)	38,364 (19.0)	
Taipei County	72,844 (13.7)	47,269 (14.3)	25,575 (12.7)	
Monthly salary (%)				0.242
High	105,751 (19.9)	55,270 (16.8)	50,481 (25.0)	
Low	164,500 (31.0)	112,847 (34.2)	51,653 (25.6)	
Middle	261,030 (49.1)	161,635 (49.0)	99,395 (49.3)	
Urbanization (%)				0.112
1 (most urbanized)	170,385 (32.1)	109,857 (33.3)	60,528 (30.0)	
2	162,331 (30.6)	102,753 (31.2)	59,578 (29.6)	
3	72,020 (13.6)	43,799 (13.3)	28,221 (14.0)	
4	64,263 (12.1)	37,738 (11.4)	26,525 (13.2)	
5 (least urbanized)	10,579 (2.0)	5648 (1.7)	4931 (2.4)	
Age (mean (SD))	52.86 (17.43)	53.33 (16.95)	52.07 (18.15)	0.072
Housing index	122.325 ± 28.33			
Yearly change	9.196 ± 7.617			

Note: SMD, standardized mean difference.

**Table 2:** Distributed lag nonlinear model analysis of the housing market and mental disorder prevalence by sex.

	Male			Female			All		
	RR^d^	Lower RR	Upper RR	RR	Lower RR	Upper RR	RR	Lower RR	Upper RR
Peak^a^	1.082	0.988	1.185	1.069	0.976	1.170	* 1.133	1.009	1.273
lag1	0.739	0.241	2.266	0.724	0.239	2.196	* 1.333	1.021	1.742
lag2	1.557	0.008	>999	1.770	0.011	291.704	0.887	0.644	1.220
lag3	0.718	0.000	>999	1.184	0.000	>999	1.112	0.896	1.380
lag4	1.156	0.014	93.726	0.632	0.009	44.292	1.022	0.962	1.086
Housing Index^b^	1.012	0.030	34.351	1.009	0.989	1.029	0.731	0.081	6.621
lag1	0.000	0.000	>999	0.649	0.405	1.039	0.000	0.000	24.105
lag2	>999	0.000	>999	6.027	0.881	41.234	>999	0.039	>999
lag3	0.000	0.000	>999	0.086	0.006	1.280	0.001	0.000	0.475
lag4	>999	0.000	>999	2.937	0.857	10.068	0.622	0.204	1.890
High Season^c^	* 1.151	1.005	1.319	* 1.379	1.213	1.567	1.121	0.980	1.282
lag1	0.351	0.007	16.598	0.064	0.002	2.273	0.961	0.039	23.738
lag2	1.233	0.000	>999	>999	0.000	>999	0.004	0.000	>999
lag3	43.967	0.000	>999	0.000	0.000	>999	>999	0.000	>999
lag4	0.041	0.000	>999	102.611	0.000	>999	0.001	0.000	>999
Linear Trend	1.019	0.968	1.072	* 1.101	1.047	1.159	* 1.433	1.302	1.577
quarterQ2	1.086	1.003	1.176	1.017	0.941	1.099	* 1.090	1.004	1.185
quarterQ3	1.104	1.012	1.206	1.061	0.973	1.156	* 1.167	1.080	1.261
quarterQ4	* 1.207	1.118	1.303	* 1.234	1.145	1.330	* 1.332	1.234	1.439
SARS^e^	0.974	0.829	1.145	1.062	0.903	1.249	1.036	0.889	1.207
Yearly Change^f^	0.991	0.970	1.011	0.977	0.958	0.997	0.995	0.982	1.008
Stock^g^	1.000	1.000	1.000	1.000	1.000	1.000	1.000	1.000	1.000
Crisis^h^	0.865	0.728	>999	0.731	0.620	0.861	0.989	0.841	1.163

^a^. Peak, local maximum of the housing index; ^b^. Housing, Taiwan Housing Index; ^c^. High season, global maximum of the housing index which is a dummy variable for housing index >100; ^d^. RR, relative risk; ^e^. SARS, severe acute respiratory syndrome outbreak period; ^f^. Yearly Change, the yearly change between two consecutive years; ^g^. The opening price of the Taiwan Stock Market; ^h^. Crisis, the financial crisis of 2008–2009; *. *p* < 0.05.

**Table 3:** Regression analysis of housing index and prescriptions prevalence by income.

	Low Income		Middle Income		High Income	
	β	*p* Value	β	*p* Value	β	*p* Value
Peak ^a^	0.171	* 0.003	0.068	0.149	0.093	0.363
lag1	0.309	0.052	0.057	0.937	−1.517	0.399
lag2	−0.001	0.997	−0.412	0.911	6.954	0.402
lag3	−0.037	0.762	0.194	0.975	−10.170	0.429
lag4	0.05	0.139	0.024	0.994	4.695	0.458
Housing Index ^b^	0.002	0.836	−0.036	0.064	−0.030	0.279
lag1	−0.052	0.211	0.567	0.203	0.559	0.385
lag2	0.036	0.418	−1.853	0.294	−1.884	0.467
lag3	−0.019	0.384	2.09	0.389	2.2	0.538
lag4	−0.003	0.467	−0.754	0.488	−0.844	0.598
High Season ^c^	0.248	* <0.001	0.187	* 0.035	−0.014	0.893
lag1	−0.113	0.769	−2.481	0.335	0.116	0.18
lag2	−0.624	0.334	9.972	0.438	0.095	0.263
lag3	0.358	0.439	−15.640	0.453	0.341	0.073
lag4	0.127	0.243	7.986	0.45	−6.747	0.144
Linear Trend	0.072	0.113	0.093	0.102	30.56	0.19
quarterQ2	0.106	* 0.030	0.129	* 0.023	−47.480	0.204
quarterQ3	0.298	* <0.001	0.086	0.085	23.39	0.211
quarterQ4	0.216	* <0.001	0.042	0.142	−0.005	0.951
SARS ^d^	0.013	0.873	0.02	0.841	−0.080	0.619
Yearly Change ^e^	−0.012	0.166	0.023	0.171	0.019	0.404
Stock ^f^	0.001	* 0.047	0.001	* 0.002	0	0.931
Crisis ^g^	−0.108	0.15	0.349	* 0.023	0.013	0.942

^a^. Peak, local maximum of the housing index. ^b^. Housing, Taiwan Housing Index. ^c^. High season, global maximum of the housing index which is a dummy variable of the housing index > 100 or not. ^d^. SARS, severe acute respiratory syndrome outbreak period. ^e^. Yearly Change, the yearly change of two consecutive year ^f^. The open price of the Taiwan Stock Market to reflect the dynamic of the stock market ^g^. Crisis, the financial crisis of 2008–2009. * *p* < 0.05.

## Data Availability

Data are available from the National Health Insurance Research Database (NHIRD) published by Taiwan National Health Insurance (NHI) Bureau. Due to legal restrictions imposed by the government of Taiwan in relation to the “Personal Information Protection Act”, data cannot be made publicly available. Requests for data can be sent as a formal proposal to the NHIRD (http://nhird.nhri.org.tw (6 March 2016)).

## Supplementary Materials

The following are available online at https://www.mdpi.com/article/10.3390/ijerph18094839/s1, Table S1: Distributed lag nonlinear model analysis of the housing market and mental disorder prevalence by administrative districts.
