# Supplementary material for: An Exploratory Study of the Association between Housing Price Trends and Antidepressant Use in Taiwan: A 10-Year Population-Based Study
Source: Int J Environ Res Public Health. 2021 Apr 30;18(9):4839. doi: 10.3390/ijerph18094839 (PMC8124140; doi:10.3390/ijerph18094839)
Supplement: Supplementary file 1 [file ijerph-18-04839-s001.zip › ijerph-1085926-supplementary.pdf]

Supplementary Table S1. Distributed lag nonlinear model analysis of the housing market and mental disorder prevalence by administrative districts.

	Taipei		Taichung		Kaohsiung	
	**β**	***p* value**	**β**	***p* value**	**β**	***p* value**
Peak[a]	−0.048	0.400	0.044	0.532	0.201	0.021
lag1	−0.307	0.748	0.379	0.679	0.159	0.898
lag2	3.513	0.443	−2.652	0.566	−1.400	0.811
lag3	−6.307	0.383	4.671	0.532	1.755	0.849
lag4	3.161	0.377	−2.407	0.520	−0.655	0.885
Housing Index[b]	0.562	0.638	0.004	0.483	1.236	0.033
lag1	−30.977	0.417	−0.091	0.605	9.924	0.503
lag2	154.992	0.401	0.381	0.656	−22.249	0.756
lag3	−244.130	0.403	−0.562	0.676	22.077	0.847
lag4	120.064	0.409	0.269	0.684	−7.674	0.894
High Season[c]	0.227	0.042	0.169	0.171	−0.111	0.207
lag1	−5.639	0.093	−4.180	0.347	−0.388	0.763
lag2	20.588	0.199	15.408	0.480	5.508	0.410
lag3	−24.162	0.336	−18.553	0.589	−11.064	0.310
lag4	8.617	0.489	6.910	0.684	5.856	0.283
quarterQ2	0.211	0.004	0.131	0.084	0.056	0.477
quarterQ3	0.250	0.003	0.157	0.163	0.046	0.605
quarterQ4	0.219	0.004	0.203	0.041	0.140	0.044
SARS[d]	−0.158	0.229	−0.109	0.390	0.005	0.970

a. Peak, local maximum of the housing index.

b. Housing Index, Taiwan Housing Index.

c. High season, global maximum of the housing index which is a dummy variable of the housing index >100 or not.

d. SARS, severe acute respiratory syndrome outbreak period.

*. $p < 0.05$
